# T817MA attenuates oxidative stress and ER stress via the HSP70–HSP90 pathway after brain ischemia *in vitro* and *in vivo*

**DOI:** 10.3389/fphar.2025.1701967

**Published:** 2025-12-03

**Authors:** Tao Wang, Ya-Juan Pan, Mei-Mei Zhang, Xuan Wang, Wei Li, Jian-Meng Lv

**Affiliations:** Department of Neurology, Shaanxi Provincial People’s Hospital, Xi’an, Shaanxi, China

**Keywords:** stroke, T817MA, oxidative stress, endoplasmic reticulum stress, HSP70, HSP90

## Abstract

Ischemic stroke imposes a substantial global burden, with oxidative stress and endoplasmic reticulum (ER) stress being the pivotal pathological mechanisms. Heat shock proteins (HSPs), especially HSP70 and HSP90, play critical roles in neuroprotection against ischemic injury. In this study, we investigate the neuroprotective effects of T817MA, a novel compound, and its underlying mechanisms *in vitro* and *in vivo*. Using oxygen–glucose deprivation (OGD)-treated HT22 cells and a middle cerebral artery occlusion (MCAO) mouse model, we found that T817MA (1 μM–3 μM) significantly attenuated OGD-induced neuronal injury, as evidenced by improved cell viability, reduced lactate dehydrogenase (LDH) release, and decreased reactive oxygen species (ROS) production. Mechanistically, T817MA suppressed ER stress by inhibiting CHOP expression and reducing intracellular Ca^2+^ release from the ER. Western blot analysis revealed that T817MA upregulated HSP70 while downregulating HSP90 in OGD-treated cells and MCAO mice. Blocking HSP70 with PES abolished T817MA-mediated protection, confirming the essential role of the HSP70–HSP90 pathway. *In vivo*, oral administration of T817MA (30 mg/kg) for 20 days prior to MCAO reduced brain edema, preserved NeuN^+^ neurons, and produced transient improvements in neurological function during the early post-ischemic period (3–5 days), although no lasting behavioral recovery was observed. Immunostaining and Western blotting showed that T817MA mitigated ER stress (i.e., reduced CHOP) and modulated HSP70/HSP90 expression in the ischemic brain. Collectively, T817MA exerts neuroprotection against brain ischemia by alleviating oxidative and ER stress via the HSP70–HSP90 signaling pathway, thus highlighting its potential as a therapeutic agent for ischemic stroke.

## 1 Introduction

Ischemic stroke, defined as cerebral blood flow interruption and subsequent neuronal damage, is a leading cause of global morbidity and mortality (Martin et al., 2025). Recent studies have elucidated the intricate molecular pathways underlying its pathophysiology, including oxidative stress, mitochondrial dysfunction, metabolic dysregulation, neuroinflammation, and multiple types of programmed neuronal death (Cheng et al., 2022; Xie et al., 2024). Advances in understanding these signaling cascades have catalyzed the development of multi-target therapies, such as ferroptosis inhibitors, metabolic modulators, and BBB protectants (Rehman et al., 2024; Tuo and Lei, 2024; Wang et al., 2024). However, more studies should be carried out in the future to prioritize combinatorial strategies to address the temporal complexity of stroke pathology, such as coupling acute neuroprotection with long-term metabolic recovery.

Heat shock proteins (HSPs) are a highly conserved family of molecular chaperones that are classified by molecular weight into HSP100, HSP90, HSP70, HSP60, HSP40, and small HSPs, including HSP27 and HSP20 (Urbanics, 2002). Functionally, HSPs maintain proteostasis via facilitating protein folding, preventing aggregation, and directing misfolded proteins for degradation through autophagy or the ubiquitin–proteasome system (Li et al., 2024). Their expression is dynamically regulated under stress conditions, such as brain ischemia, hemorrhage, oxidative stress, and inflammation, making them critical mediators of cellular survival and injury resolution (Sharp and Jickling, 2013; Shao et al., 2019). In ischemic stroke, HSP70 exerts neuroprotection by inhibiting mitochondrial apoptosis via Bcl-2 upregulation and caspase-3 suppression while concurrently stabilizing the blood–brain barrier (BBB) through matrix metalloproteinase-9 (MMP-9) inhibition (Doeppner et al., 2017). HSP32 attenuates oxidative stress by metabolizing heme into biliverdin and carbon monoxide, although its iron-releasing activity may paradoxically exacerbate ferroptosis in prolonged ischemia (Shah et al., 2011; Wang et al., 2016). Small HSPs, such as HSP27, modulate the vascular tone by suppressing cytoskeletal destabilization and endothelial contraction, thereby reducing cerebral vasospasm (Knoepp et al., 2000; Stetler et al., 2012). Previous studies have suggested several potential protective agents targeting HSPs for stroke therapy, such as HSP inhibitors and traditional Chinese medicine compounds.

T817MA, also known as edonerpic maleate, is a recently synthesized compound with neurotrophic potential to the central nervous system (Hirata et al., 2005). It was originally screened based on the neuroprotective potency against Aβ-induced neurotoxicity, with the neurite outgrowth-promoting effect. In cultured cortical neurons, T817MA was shown to protect against mitochondrial dysfunction induced by the NO donor sodium nitroprusside via a newly synthesized protein-mediated mechanism (Fukushima et al., 2006). It was found to enhance the experience-driven delivery of the synaptic glutamate AMPA receptors and accelerate rehabilitation following motor cortex cryoinjury in mice by cortical reorganization (Abe et al., 2018). In addition, T817MA has been demonstrated to have therapeutic potential in experimental traumatic brain injury (TBI) models by regulating the collapsing response mediator protein 2 (CRMP2) and activity-regulated cytoskeletal (Arc) expression (Chen et al., 2022). However, the effect of T817MA on neuronal death and neurological dysfunction following brain ischemia remains unknown. In the present study, we investigated the effect of T817MA on brain damage caused by transient middle cerebral artery occlusion (MCAO) and the potential underlying mechanisms, with a focus on HSP signaling. In this study, we focused on the novel effects of T817MA on endoplasmic reticulum (ER) stress and the HSP70–HSP90 chaperone pathway during ischemic injury. We provide the first evidence that T817MA attenuates ER stress, as indicated by the reduction in CHOP expression and the suppression of ER calcium release. Additionally, we show that T817MA regulates HSP70 and HSP90, which are critical components of cellular protein homeostasis and stress responses, likely contributing to its neuroprotective effects against ischemic brain injury. These findings represent a novel mechanism for the neuroprotective action of T817MA in ischemic stroke and offer insights into its potential as a therapeutic agent targeting ER stress and the protein quality-control machinery.

## 2 Materials and methods

### 2.1 Ethics and animals

Male adult C57BL/6 mice (weight: 25 g–30 g) were housed in a regular 12 h light/dark cycle with free access to food and water at a relatively constant temperature (approximately 22 °C). The study was performed in accordance with the Guide for the Care and Use of Laboratory Animals of the National Institutes of Health and was reported in compliance with the ARRIVE guidelines.

### 2.2 Culture of HT22 cells

The immortalized mouse hippocampal neurons, HT22 cells, were obtained from the Bena Culture Collection located in China. Cells were maintained in Dulbecco’s Modified Eagle’s Medium (DMEM; Gibco, Cat# 11965092) supplemented with 10% heat-inactivated fetal bovine serum (FBS; Gibco, Cat# 10437028) and 1% penicillin–streptomycin (100 U/mL penicillin and 100 μg/mL streptomycin; Gibco, Cat# 15140122). Cells were incubated at 37 °C in a humidified atmosphere of 5% CO_2_/95% air. Cultures were passaged every 2–3 days at 70%–80% confluence.

### 2.3 *In vitro* ischemia model

The *in vitro* ischemia model was established using the oxygen–glucose deprivation (OGD) method. For OGD induction, the medium was replaced with glucose-free DMEM, and the cultures were transferred to a hypoxic chamber flushed with 5% CO_2_ and 95% N_2_ for 4 h at 37 °C. Reoxygenation was initiated by replacing the medium with normal complete DMEM and returning the cultures to normoxic conditions. The control group was cultured according to the routine procedures during this period.

### 2.4 Calcein signal measurement

Cell viability of HT22 cells after OGD was determined by measuring the calcein-AM signal using a kit according to the manufacturer’s protocol (Enzo Life Sciences, Farmingdale, NY, USA).

### 2.5 LDH release measurement

Neuronal injury *in vitro* was detected by measuring lactate dehydrogenase (LDH) release into the culture medium using a commercial kit according to the manufacturer’s protocol (Jian-Cheng Biotec, Nanjing, China).

### 2.6 Measurement of ROS generation

Intracellular ROS levels in HT22 cells were quantified using the oxidant-sensitive probe 2′-7′-dichlorodihydrofluorescein diacetate (DCFH-DA; abs42197174, Sigma). Post-OGD, HT22 cells were washed twice with phosphate-buffered saline (PBS) and incubated with 10 μM DCFH-DA diluted in serum-free DMEM (pH 7.0) for 30 min at 37 °C in the dark. A positive control group was treated with 100 μM Rosup (abs580232, SANS Biological) for 30 min to induce ROS generation, and a negative control received serum-free DMEM alone. ROS-dependent oxidation of DCFH to fluorescent 2′,7′-dichlorofluorescein (DCF) was detected by measuring DCF fluorescence (green) using a Zeiss LSM 880 confocal microscope (488-nm excitation, 500–550-nm emission).

### 2.7 Ca^2+^ imaging

Ca^2+^ imaging was performed using the ratiometric indicator Fura-2 AM (5 μM) dissolved in Hank’s Balanced Salt Solution (HBSS) containing 0.02% pluronic F-127 to enhance dye dispersion and cellular uptake. HT22 cells cultured on glass coverslips were incubated with the loading solution for 30 min at room temperature under light-protected conditions, followed by a 30-min lucifugal equilibration period in dye-free HBSS to facilitate complete de-esterification of intracellular Fura-2 AM into active Fura-2. Ratiometric imaging was conducted using a confocal laser scanning microscope equipped with dual-excitation filters (340 nm and 380 nm) and an emission filter (510 nm). The 340/380 nm excitation ratio was calculated to quantify changes in intracellular calcium concentration ([Ca^2+^]_cyt_), minimizing artifacts from uneven dye distribution, photobleaching, or cell thickness variations. Baseline fluorescence (F_0_) was determined by averaging the initial 50 s of recordings under resting conditions. Data normalization was expressed as F/F_0_, where F represents the real-time ratio. Quantitative analysis included calculating the area under the curve (AUC) and peak F/F_0_ values to evaluate the cumulative calcium influx and transient amplitudes, respectively. All Ca^2+^ imaging experiments were performed under calcium-free conditions. During recordings, cells were bathed in Ca^2+^-free HBSS to eliminate extracellular calcium influx, ensuring that the Fura-2 AM fluorescence changes primarily reflected intracellular Ca^2+^ release. This setup allowed us to specifically assess Ca^2+^ mobilization from intracellular stores, particularly the ER, during OGD or Tg treatment.

### 2.8 Middle cerebral artery occlusion (MCAO)

Brain ischemia was induced in mice using the intraluminal filament technique, as previously described, with modifications. Briefly, animals were anesthetized with 5% isoflurane in oxygen and maintained at 2%–2.5% during surgery. Following a midline neck incision, the right common carotid artery (CCA), external carotid artery (ECA), and internal carotid artery (ICA) were carefully exposed. A silicone-coated 6–0 monofilament (diameter: 0.22 ± 0.01 mm; coating length: 3 mm–4 mm) was introduced into the ECA lumen and advanced till 9 mm–10 mm rostral through the ICA until mild resistance indicated the occlusion of the middle cerebral artery (MCA) origin. The MCA was embolized for 60 min to induce ischemia, followed by the removal of the monofilament. Sham-operated mice underwent identical procedures without filament insertion. Mice were placed in a thermostatic blanket to maintain a constant anal temperature throughout the procedure.

### 2.9 Measurement of brain edema

Brain water content was quantified using the gravimetric method to determine brain edema. Mice were euthanized under deep anesthesia (5% isoflurane) at predetermined time points post-injury. The brains were rapidly extracted (<2 min) and divided into ipsilateral and contralateral hemispheres using a rodent brain matrix. Tissue samples were immediately weighed on precooled aluminum foil to obtain their wet weight (WW) using an analytical balance. Samples were then dehydrated in an oven at 105 °C for 72 h until constant weight was achieved, which was confirmed by three consecutive stable measurements at 24-h intervals. The dry weight (DW) was recorded, and brain water content (%) was calculated using the formula: [(WW - DW)/WW] × 100.

### 2.10 Neurological dysfunction assessment

The rotarod test was utilized to evaluate motor coordination, balance, and fatigue resistance in mice following MCAO, using an SA102 rotarod apparatus (SANS Biological Technology, Nanjing, China) equipped with a 30-mm-diameter rod for mice and an acceleration control module. Preoperative training was conducted over 5 days. For the first 2 days, mice were acclimatized to a static rod for 5 min, followed by low-speed rotation (constant 4 rpm) for 10 min/day. For the last 3 days, gradual acceleration protocols were introduced (4 rm–20 rpm over 2 min), with three trials/day interspersed by 30-min rest intervals to prevent exhaustion. Animals failing to remain on the rod for >60 s after three attempts were excluded to ensure cohort uniformity. Postoperative testing commenced at 1, 3, 5, 7, and 14 days after MCAO. Each session comprised three trials (15-min intervals) under an accelerated regimen (4 rpm–40 rpm over 5 min, 7.2 rpm/min acceleration). The maximum trial duration was capped at 300 s to mitigate stress.

### 2.11 Immunohistochemistry

Coronal sections (20 μm) from paraformaldehyde-perfused brains were collected using a cryostat (Leica CM1950) at −20 °C. After antigen retrieval in citrate buffer (pH 6.0, 95 °C, 20 min), sections were blocked with 10% normal goat serum in 0.3% Triton X-100/PBS for 1 h at room temperature (RT). Primary antibody incubation with anti-NeuN (1:200, ab177487) or anti-CHOP (1:100, ab11419) was performed overnight at 4 °C in a humidified chamber. Following three PBS washes (5 min each), sections were incubated with Alexa Fluor 594- or 488-conjugated IgG (1:1000, Invitrogen, A-11005) for 2 h at RT. Nuclei were counterstained with DAPI (5 μg/mL, Sigma, 5 min), and slides were mounted with anti-fade medium. Fluorescence images were captured using a confocal microscope (Zeiss LSM 900) with identical exposure settings.

### 2.12 Western blot

Equal amounts of protein (30 μg/lane) were separated on 10% SDS-PAGE gels and transferred to 0.45 μm PVDF membranes at 300 mA for 90 min. Membranes were blocked with 5% nonfat milk in TBST (Tris-buffered saline with 0.1% Tween-20) for 1 h at RT and then incubated overnight at 4 °C with the following primary antibodies: anti-CHOP (1:800, ab11419), anti-HSP70 (1:500, sc-59569), anti-HSP90 (1:600, sc-69703), and anti-β-actin (1:5000, Sigma, A5441). After three TBST washes (10 min each), membranes were probed with HRP-conjugated secondary antibodies for 1 h at RT. Protein bands were visualized using the chemiluminescent detection system.

### 2.13 Statistical analysis

Statistical analysis was performed using SPSS 16.0. Statistical evaluation of the data was performed by one-way analysis of variance (ANOVA), followed by Bonferroni’s multiple comparisons.

## 3 Results

### 3.1 T817MA protects against OGD-induced neuronal injury *in vitro*

**FIGURE 1:**
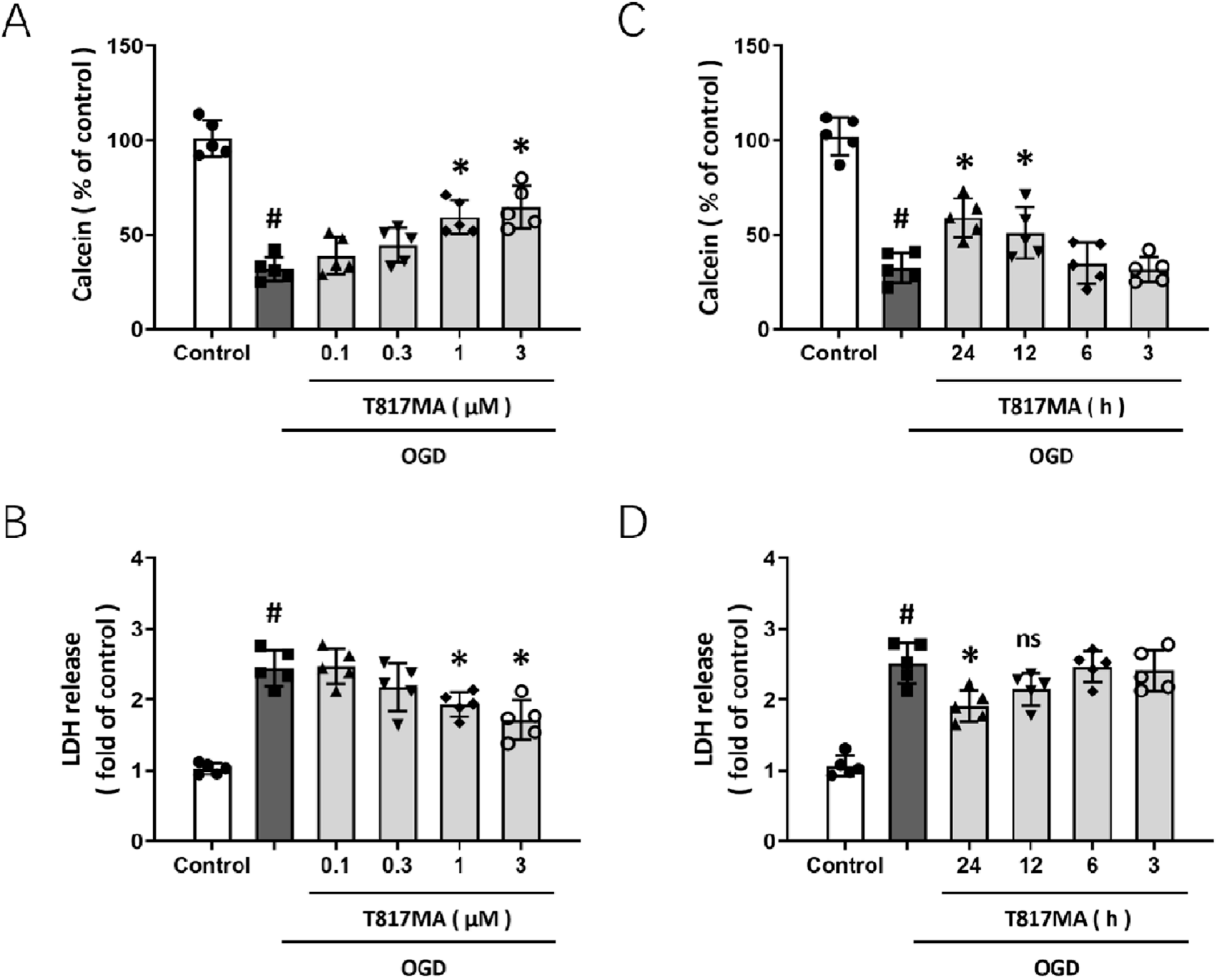
T817MA protects against OGD-induced neuronal injury *in vitro*. **(A)** T817MA at 1 and 3 μM preserves cell viability after OGD in HT22 cells. **(B)** T817MA at 1 and 3 μM decreases LDH release after OGD. **(C)** Pretreatment with 1 μM of T817MA for 24 and 12 h preserves cell viability after OGD. **(D)** Pretreatment with 1 μM T817MA for 24 and 12 h decreases LDH release after OGD. n = 5 in each group. Data are shown as the mean ± SD. ^#^ *p* < 0.05 vs. the control group. ^*^ *p* < 0.05 vs. the OGD group.

To investigate the effect of T817MA on neuronal injury following OGD, HT22 cells were pretreated with T817MA at different concentrations for 24 h, and cell viability was assessed by measuring the calcein signal (Figure 1A). The results showed that OGD significantly reduced calcein signal, which was attenuated by 1 and 3 μM T817MA but not by 0.1 and 0.3 μM T817MA. As shown in Figure 1B, similar results in the LDH release assay were also observed. To further determine the therapeutic time window of T817MA, it was pretreated at different points before OGD, and then the calcein signal (Figure 1C) and LDH release assays (Figure 1D) were performed. It was found that pretreatment with 1 μM T817MA for 24 and 12 h, but not for 6 or 3 h, attenuated the OGD-induced decrease in calcein signal (Figure 1C). However, only 24-h pretreatment of T817MA exerted a protective effect against OGD-induced LDH release in HT22 cells (Figure 1D).

### 3.2 T817MA attenuates OGD-induced oxidative stress and ER stress

**FIGURE 2:**
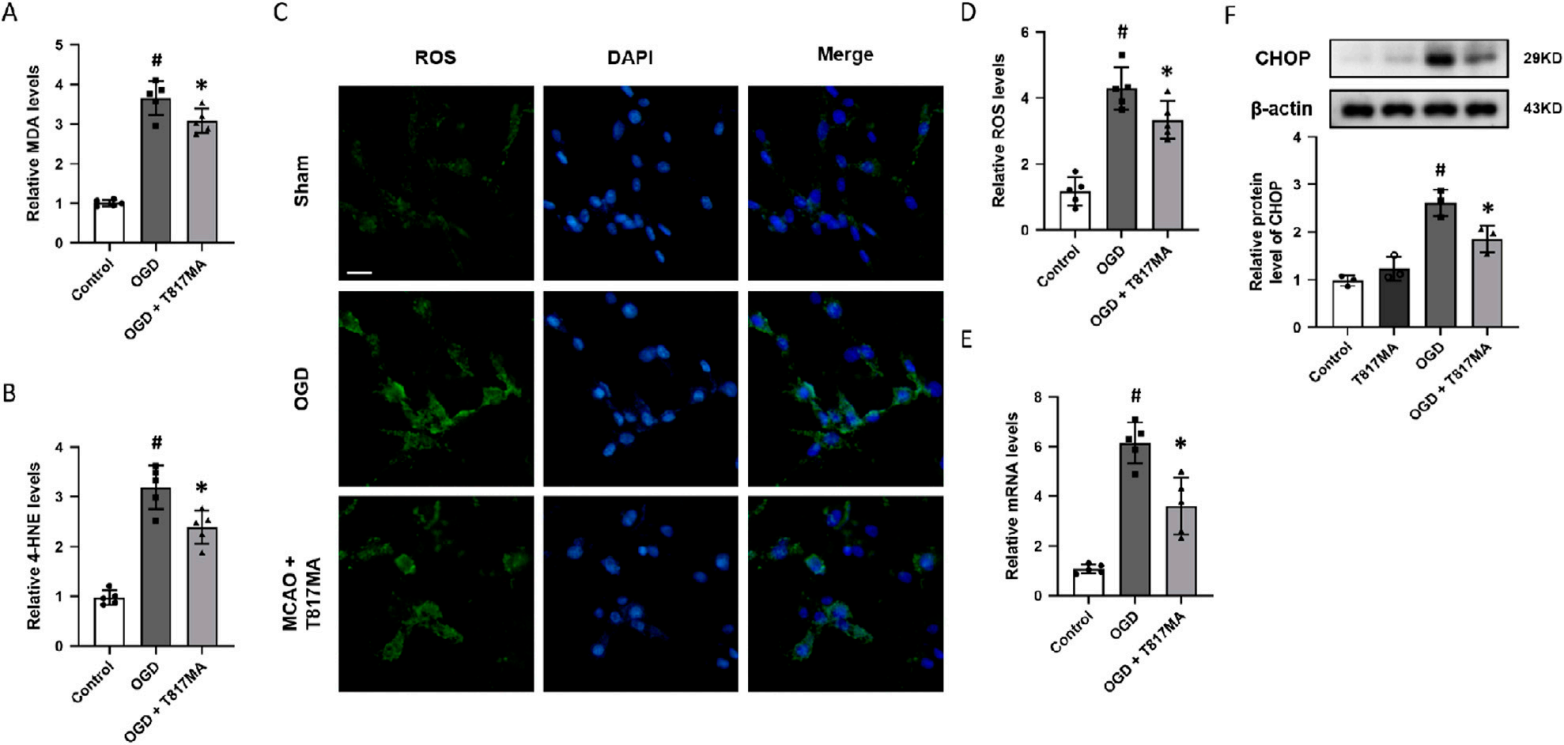
T817MA attenuates OGD-induced oxidative stress and ER stress. **(A)** T817MA decreases MDA levels after OGD. **(B)** T817MA decreases 4-HNE levels after OGD. **(C, D)** Immunostaining **(C)** and quantitative analysis **(D)** show that T817MA decreases intracellular ROS generation after OGD. **(E)** RT-PCR analysis shows that the OGD-induced increase in the PERK mRNA level is reduced by T817MA. **(F)** Western blot analysis shows that the OGD-induced expression of CHOP is attenuated by T817MA (scale bar = 10 μm). n = 5 **(A–E)** and n = 3 **(F)** in each group. Data are shown as the mean ± SD. ^#^ *p* < 0.05 vs. the control group. ^*^ *p* < 0.05 vs. the OGD group.

To investigate the effect of T817MA on oxidative stress, the relative MDA (Figure 2A) and 4-HNE (Figure 2B) levels were measured to determine lipid peroxidation. The results showed that the OGD-induced increases in these two factors were both reduced by T817MA. Immunostaining with the oxidant-sensitive probe DCFH-DA was performed to detect the intracellular ROS generation after OGD in HT22 cells (Figure 2C), and we found that the increased ROS levels induced by OGD were attenuated by T817MA (Figure 2D). The mRNA level of PERK was increased by OGD but decreased by T817MA (Figure 2E). In addition, the results of the Western blot analysis showed that the expression of the CHOP protein was elevated after OGD in HT22 cells, indicating the induction of ER stress (Figure 2F). T817MA reduced CHOP expression following OGD, but it had no effect on CHOP expression in control cells.

### 3.3 T817MA inhibits intracellular ER Ca^2+^ release in HT22 cells

**FIGURE 3:**
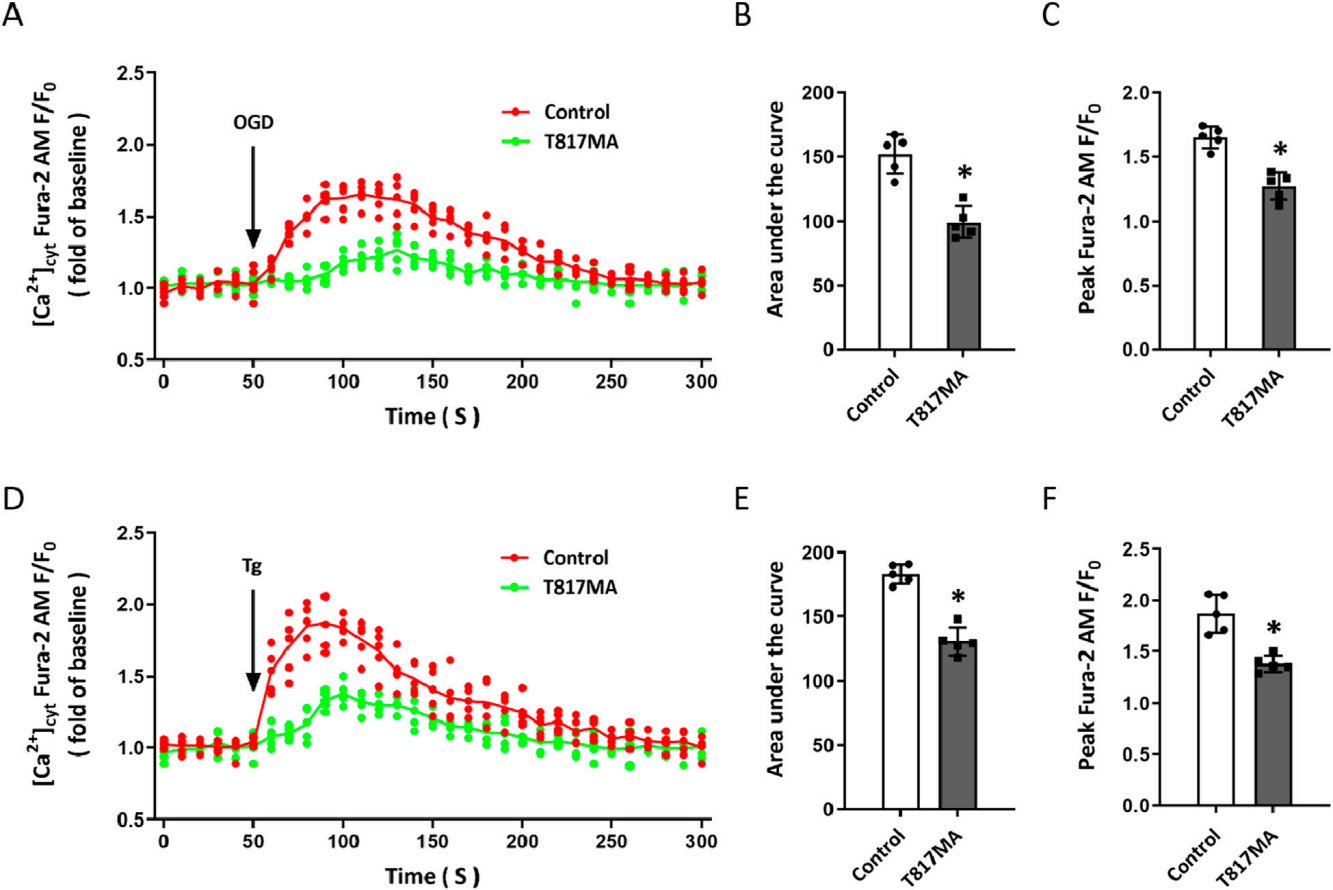
T817MA inhibits intracellular ER Ca^2+^ release in HT22 cells. **(A–C)** Ca^2+^ imaging **(A)** and quantitative analysis **(B,C)** show that T817MA reduces OGD-induced Ca^2+^ release **(B)** and decreases the peak intracellular Ca^2+^ level **(C)** in HT22 cells. **(D–F)** Ca^2+^ imaging **(D)** and quantitative analysis **(E,F)** show that T817MA reduces Tg-induced Ca^2+^ release **(E)** and decreases the peak intracellular Ca^2+^ level **(F)** in HT22 cells. n = 5 in each group. Data are shown as the mean ± SD. ^*^ *p* < 0.05 vs. the control group.

ER plays a key role in intracellular Ca^2+^ homeostasis. To investigate the effect of T817MA on intracellular Ca^2+^ regulation in HT22 cells, OGD (Figure 3A) and the selective sarco/endoplasmic reticulum Ca^2+^-ATPase (SERCA) inhibitor Tg (Figure 3D) were used to perform Ca^2+^ imaging before T817MA treatment. The results showed that OGD-induced Ca^2+^ increase, as evidenced by AUC (Figure 3B) and the peak F/F0 values (Figure 3C), was markedly reduced by T817MA. As shown in Figures 3E,F, similar results were also observed after adding Tg, which indicated the involvement of ER Ca^2+^ release.

### 3.4 T817MA regulates the HSP70–HSP90 pathway following OGD

**FIGURE 4:**
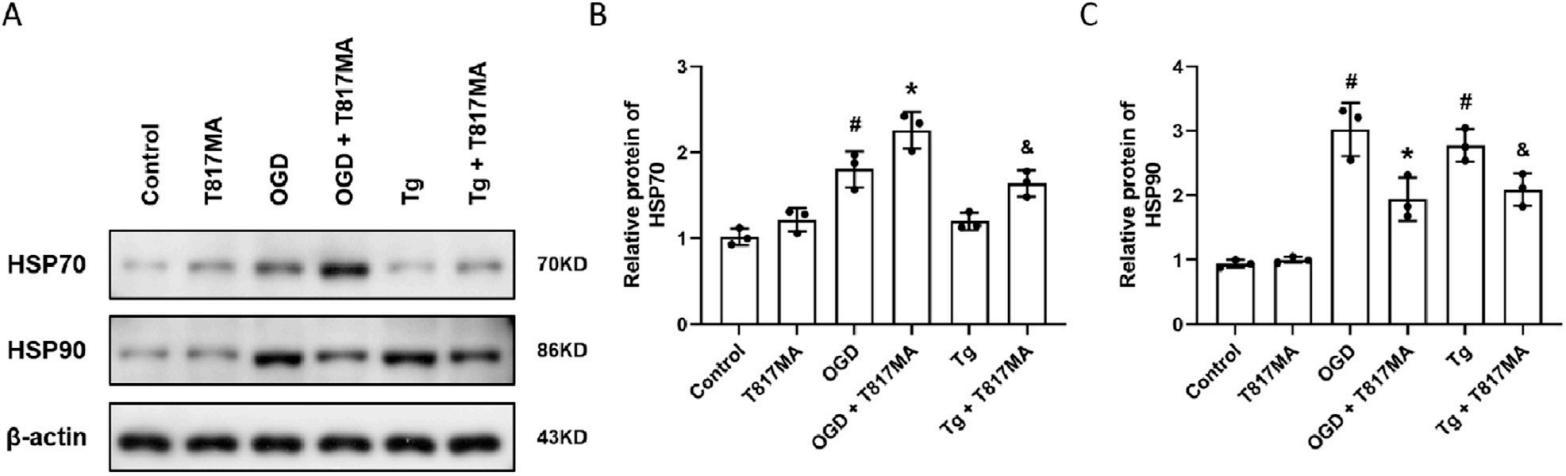
T817MA regulates the HSP70–HSP90 pathway following OGD. **(A–C)** Western blot **(A)** and quantitative analyses **(B,C)** show that T817MA enhances the expression of HSP70 following OGD and Tg **(B)**, but T817MA decreases the expression of HSP90 following OGD and Tg **(C)**. n = 3 in each group. Data are shown as the mean ± SD. ^#^ *p* < 0.05 vs. control group. ^*^ *p* < 0.05 vs. the OGD group. ^&^ *p* < 0.05 vs. the Tg group.

To investigate the effects of T817MA on the HSP70–HSP90 pathway, the Western blot analysis was performed (Figure 4A). OGD significantly increased HSP70 expression, whereas T817MA or Tg had no effect on HSP70 expression (Figure 4B). However, T817MA further enhanced HSP70 expression in OGD- and Tg-treated cells. As shown in Figure 4C, OGD and Tg markedly increased the expression of HSP90, and both were attenuated by T817MA.

### 3.5 The HSP70–HSP90 pathway contributes to T817MA-induced protection

**FIGURE 5:**
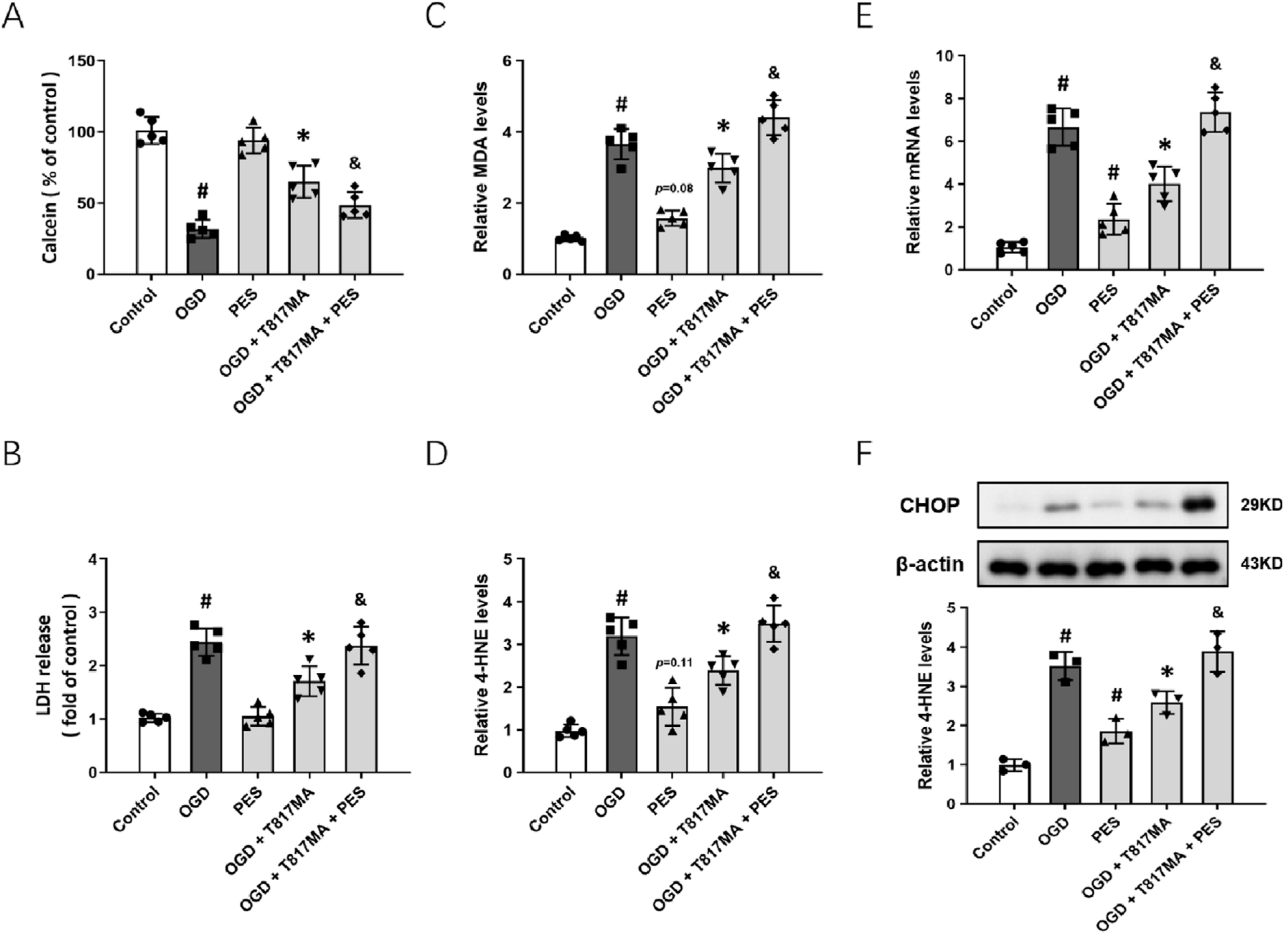
HSP70–HSP90 pathway contributes to T817MA-induced protection. **(A)** PES decreases cell viability in T817MA-treated cells following OGD. **(B)** PES increases LDH release in T817MA-treated cells following OGD. **(C)** PES increases the MDA levels in T817MA-treated cells following OGD. **(D)** PES increases 4-HNE levels in T817MA-treated cells following OGD. **(E)** RT-PCR analysis shows that PES increases the PERK mRNA levels in T817MA-treated cells following OGD. **(F)** Western blot analysis shows that PES increases CHOP expression in T817MA-treated cells following OGD. n = 5 **(A–E)** and n = 3 **(F)** in each group. Data are shown as the mean ± SD. ^#^ *p* < 0.05 vs. the control group. ^*^ *p* < 0.05 vs. the OGD group. ^&^ *p* < 0.05 vs. the OGD + T817MA group.

To confirm the involvement of the HSP70–HSP90 pathway in T817MA-induced protection *in vitro*, we repeated the above experiments after pretreatment with the HSP70 inhibitor PES. The results of the calcein signal assay showed that PES had no effect on cell viability, but it partially prevented the T817MA-induced decrease in cell viability (Figure 5A). PES also increased the LDH release in the T817MA-treated group after OGD (Figure 5B). In addition, similar results in MDA (Figure 5C) and 4-HNE (Figure 5D) levels were also observed. As shown in Figure 5E, PES increased the mRNA level of PERK in the T817MA-treated group after OGD. Furthermore, we repeated the Western blot analysis, and PES alone significantly increased CHOP expression in HT22 cells (Figure 5F). In summary, the T817MA-induced decrease in CHOP expression after OGD was reversed by PES (Figure 5E).

### 3.6 T817MA exerts neuroprotection against MCAO-induced brain ischemia

**FIGURE 6:**
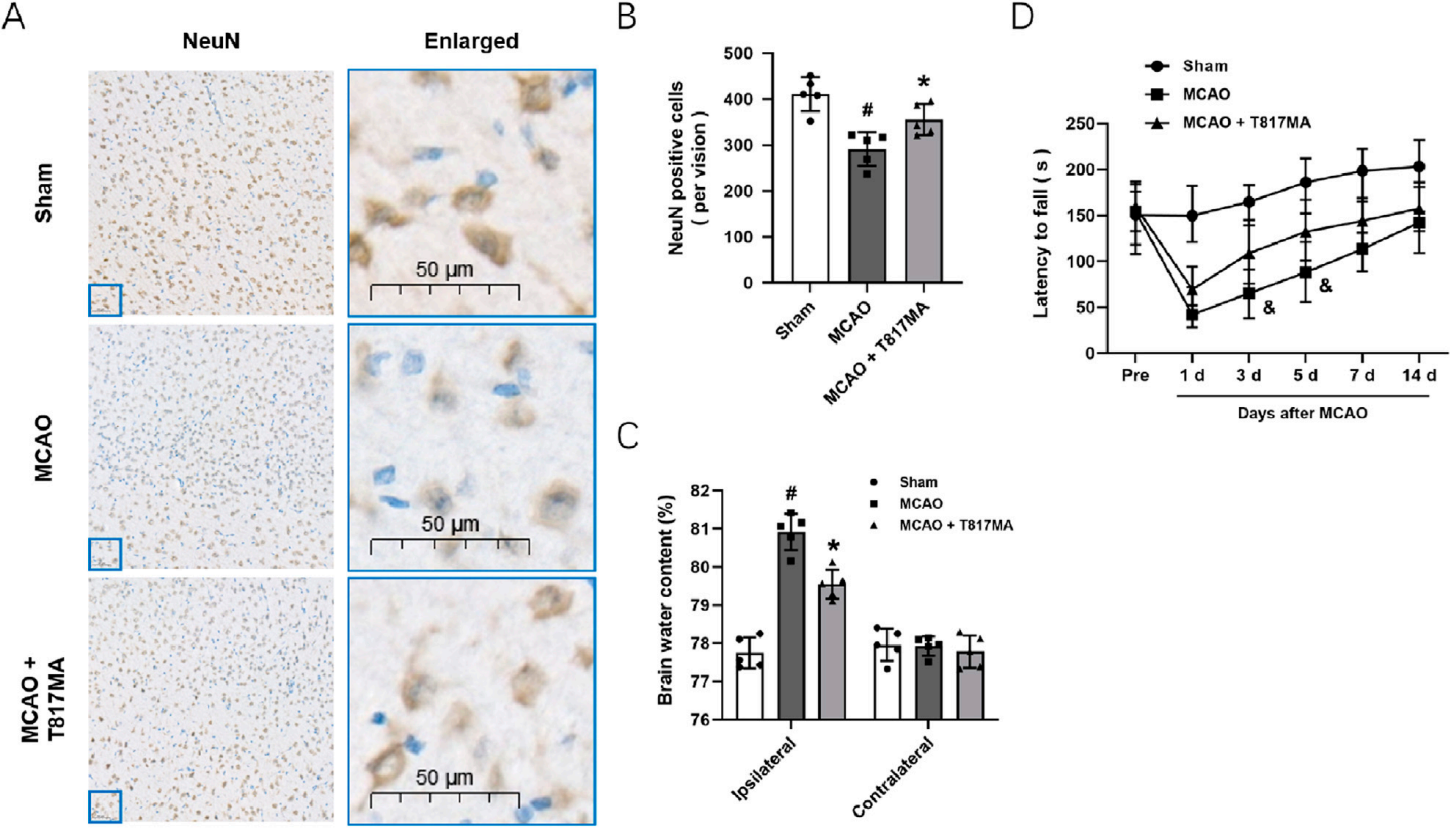
T817MA exerts neuroprotection against MCAO-induced brain ischemia. **(A,B)** Immunostaining **(A)** and quantitative analysis **(B)** show that T817MA reduces neuronal loss after MCAO in mice. **(C)** T817MA reduces brain water content after MCAO. **(D)** T817MA attenuates neurological dysfunction after MCAO (scale bar = 50 μm). n = 5 in each group. Data are shown as the mean ± SD. ^#^ *p* < 0.05 vs. the sham group. ^*^ *p* < 0.05 vs. the MCAO group. ^&^ *p* < 0.05 vs. the MCAO group.

To investigate the effects of T817MA on brain ischemia, animals were orally treated with 30 mg/kg T817MA for 20 days before MCAO, and this dose was chosen as previously published (Chen et al., 2023). Immunostaining was performed to detect the neuronal loss *in vivo* (Figure 6A), and the results showed that the decreased number of NeuN-positive cells was increased by T817MA (Figure 6B). The brain water content was measured to detect brain edema, and we found that the increased brain water content in the cerebrum induced by MCAO was attenuated by T817MA (Figure 6C). In addition, the MCAO-induced neurological dysfunction at 3 and 5 days after MCAO, but not at 1, 7, and 14 days, was attenuated by T817MA (Figure 6D).

### 3.7 T817MA regulates ER stress and the HSP70–HSP90 pathway *in vivo*

**FIGURE 7:**
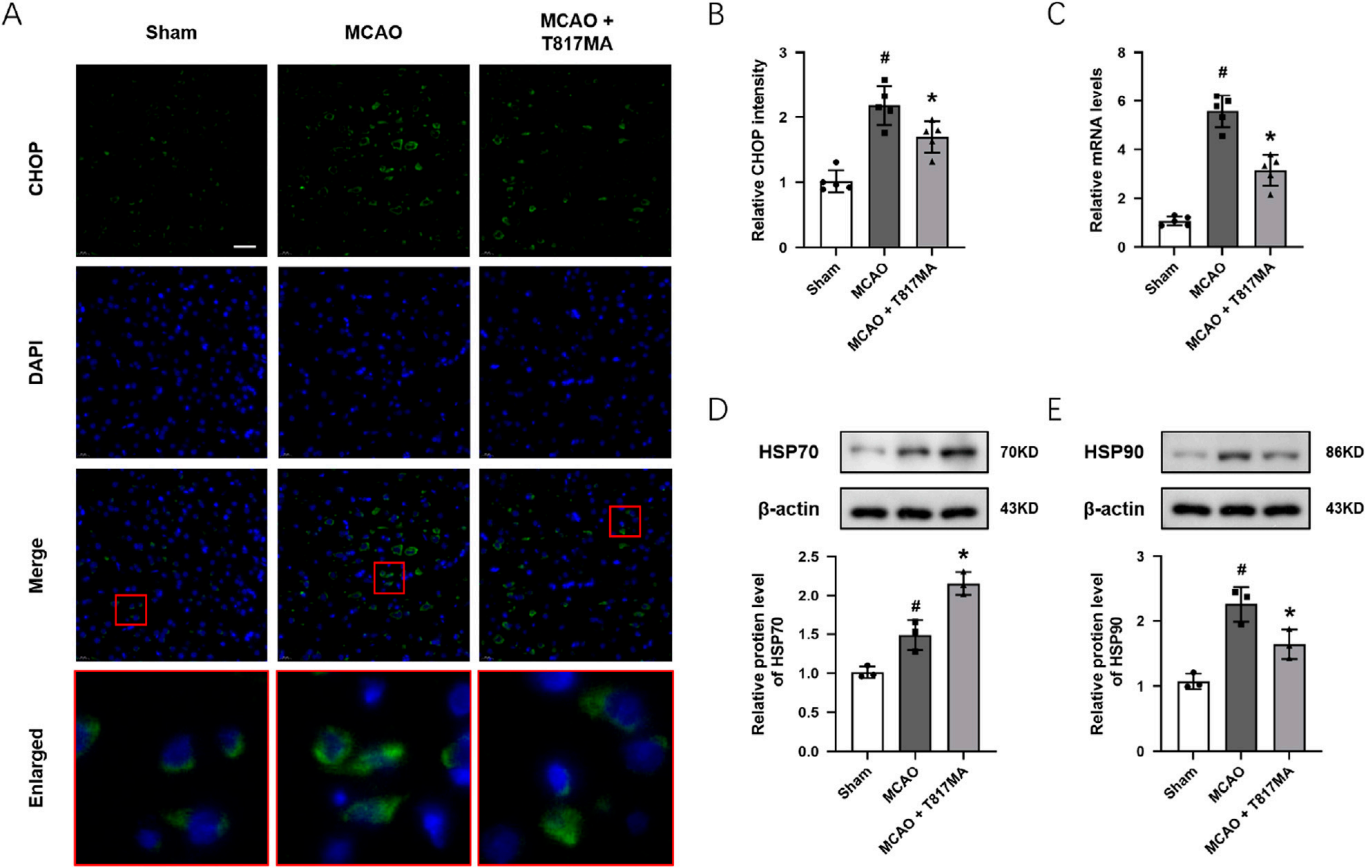
T817MA regulates ER stress and the HSP70–HSP90 pathway *in vivo*. **(A,B)** Immunostaining **(A)** and quantitative analysis **(B)** show that T817MA reduces CHOP expression after MCAO. **(C)** RT-PCR analysis shows that T817MA decreases the PERK mRNA level after MCAO. **(D)** T817MA enhances HSP70 expression after MCAO. **(E)** T817MA decreases HSP90 expression after MCAO (scale bar = 20 μm). n = 5 **(A–C)** and n = 3 **(D,E)** in each group. Data are shown as the mean ± SD. ^#^ *p* < 0.05 vs. the sham group. ^*^ *p* < 0.05 vs. the MCAO group.

To investigate the effect of T817MA on ER stress *in vivo*, immunostaining was performed to detect the expression of CHOP in brain sections after MCAO (Figure 7A). We observed that the CHOP protein showed more expression in the nucleus of neuronal cells after MCAO. The results showed that the MCAO-induced increase in CHOP fluorescence intensity was decreased by T817MA (Figure 7B). The results of RT-PCR showed that the MCAO-induced increase in the PERK mRNA level was attenuated by T817MA (Figure 7C). In addition, T817MA further enhanced HSP70 expression after MCAO (Figure 7D), but it reduced HSP90 expression following MCAO (Figure 7E).

## 4 Discussion

ER stress represents a pivotal mechanism in the pathophysiology of ischemic stroke, driving neuronal death through the dysregulation of proteostasis and the activation of multiple cell-death pathways (Prentice et al., 2015). In the present study, we identified T817MA as a neuroprotective agent against ischemic stroke-induced ER stress in both *in vitro* and *in vivo* models. We found the following: (a) T817MA attenuates neuronal injury and oxidative stress following OGD in HT22 cells, (b) T817MA inhibits intracellular ER Ca^2+^ release and CHOP expression, (c) T817MA exerts protective effects through the HSP70–HSP90 pathway following OGD, (d) T817MA reduces brain edema and preserves neurological function following brain ischemia, and (e) T817MA regulates ER stress and the HSP70–HSP90 pathway *in vivo*.

Following cerebral ischemia, disruptions in calcium homeostasis, oxidative stress, and energy depletion induce the accumulation of unfolded or misfolded proteins within the ER lumen. This triggers the unfolded protein response (UPR), which aims to restore the ER function by attenuating protein translation and upregulating chaperones such as GRP78 (Wang et al., 2022; Gebert et al., 2023). However, prolonged ischemia converts this response into a proapoptotic signal. The PERK–ATF4–CHOP axis is particularly critical, as it upregulates proapoptotic factors (e.g., Bim and Bax) and suppresses antiapoptotic proteins (e.g., Bcl-2), culminating in caspase-12 and caspase-3 activation (Oyadomari and Mori, 2004). Notably, in rodent models, CHOP expression peaks during reperfusion, coinciding with neuronal apoptosis and infarct expansion. Thus, we detected the expression of CHOP to investigate the involvement of ER stress-associated cell death in our results. In HT22 cells, the results of the Western blot analysis showed that OGD significantly increased the expression of CHOP, which was attenuated by T817MA. In addition, the immunostaining in brain sections showed that MCAO markedly increased the expression of CHOP protein and its aggregation in the nucleus of neuronal cells. Targeting ER stress has emerged as a promising neuroprotective strategy. Pharmacological inhibitors of UPR branches, such as PERK inhibitors (e.g., GSK2606414), have shown efficacy in reducing the infarct volume and improving functional recovery in preclinical models by suppressing CHOP-mediated apoptosis (Dhir et al., 2023; Quan et al., 2025). Biological agents such as neuroserpin, a serine protease inhibitor, alleviate ischemia–reperfusion injury by normalizing ER stress sensors and inhibiting downstream apoptotic effectors, including ATF4 and CHOP, in mouse MCAO models (Zhang et al., 2002; Wu et al., 2010). Similarly, our results showed that both the *in vitro* and *in vivo* ischemia-induced activation of CHOP were decreased by T817MA, which was accompanied by attenuated brain damage and neuronal injury. All these data strongly indicated that the T817MA-induced protection was, at least partially, mediated by the inhibition of ER-stress-related neuronal cell death.

Maintaining ER Ca^2+^ homeostasis is critical for neuronal proteostasis and survival (Krebs et al., 2015). The ER serves as the primary intracellular Ca^2+^ reservoir, and its depletion, triggered by genetic mutations or environmental stressors, activates UPR and induces ER stress (Chernorudskiy and Zito, 2017). In Wolfram syndrome, mutations in *WFS1* or *CISD2* disrupt ER Ca^2+^ handling, impairing IP_3_R-mediated Ca^2+^ transfer to mitochondria. This compromises mitochondrial Ca^2+^ uptake, suppresses ATP production, elevates the NADH/NAD^+^ ratio, and induces bioenergetic deficits coupled with reductive stress, culminating in neuronal degeneration (Liiv et al., 2024). Similarly, disrupted ER–mitochondrial contact sites in these models highlight how ER Ca^2+^ dysregulation propagates metabolic dysfunction. Thus, we measured intracellular Ca^2+^ levels in Ca^2+^-free medium to detect intracellular Ca^2+^ release, which partially reflects the ER Ca^2+^ homeostasis *in vitro*. The selective inhibitor of SERCA, Tg, depletes ER Ca^2+^ stores in neuronal cells by blocking Ca^2+^ reuptake into the ER, thereby disrupting ER Ca^2+^ homeostasis (Thastrup et al., 1990; Giorgi et al., 2010). Our results showed that both OGD and Tg increased intracellular Ca^2+^ levels in HT22 cells, which were attenuated by T817MA treatment, thus indicating the role of T817MA in ER Ca^2+^ regulation. Under ER stress conditions, activated CHOP further exacerbates Ca^2+^ efflux via ERO1α, amplifying mitochondrial permeability transition and cytochrome c-mediated apoptosis (Varone et al., 2021; Roy et al., 2024). Emerging approaches, including gene editing to correct mutations and engineered stem cell-derived neurons with reinforced ER homeostasis, require clinical translation validation.

Among the HSP family proteins, HSP70 and HSP90 serve as critical molecular chaperones that mitigate proteotoxic stress in ischemic stroke (Genest et al., 2019; Gupta et al., 2020). These proteins orchestrate cellular defense mechanisms against ischemia–reperfusion injury, although their individual roles and interactions exhibit distinct mechanistic profiles. HSP70 confers robust neuroprotection during ischemic stroke by stabilizing unfolded proteins, inhibiting apoptotic pathways, and attenuating neuroinflammation. In rodent models of focal cerebral ischemia, HSP70 mRNA and protein expression surge significantly upon reperfusion, peaking within 24 h–72 h (Currie et al., 2000; Zhao et al., 2006). This upregulation correlates with reduced infarct volumes and improved neurological outcomes. Pharmacological agents such as lidocaine amplify this effect, enhancing HSP70 expression and subsequently diminishing neuronal necrosis and edema (Xia et al., 2017; Cheng et al., 2019; Zhang et al., 2022). Similarly, our present data showed that the HSP70 inhibitor PES significantly aggravated OGD-induced cell death in HT22 cells. In addition, T817MA-induced protection was dampened by PES, indicating the protective role of HSP70 in our experiments. In contrast, HSP90 exhibits a more complex duality in ischemia. Although it stabilizes client proteins that are essential for cell survival, its overexpression can paradoxically sustain pathogenic signaling. Previous studies have shown that HSP90 not only stabilizes pro-survival kinases such as Akt but also promotes necroptosis via RIPK1–RIPK3–MLKL signaling, which highlights context-dependent therapeutic implications (Jacobsen et al., 2016; Xu et al., 2025). Recent advances highlight intricate cooperation between HSP70 and HSP90 in proteostasis. HSP70 acts as a primary “holder” for misfolded proteins, transferring clients to HSP90 for further refolding or degradation (Baindur-Hudson et al., 2015). Dysregulation of either chaperone disrupts this axis: reduced HSP70 impairs the initial protein capture, whereas HSP90 inhibition blocks downstream processing (Baindur-Hudson et al., 2015; Genest et al., 2019). We observed increased expression of HSP70 and HSP90 after OGD, but they were differently regulated by T817MA. The activation of HSP70 was further enhanced by T817MA, whereas T817MA markedly inhibited HSP90 expression after OGD. In addition, the HSP70 inhibitor PES reversed the expression of HSP90. Thus, HSP70 and HSP90 collectively form a dynamic defense network against ischemic proteotoxicity. Whereas HSP70 predominantly mitigates acute damage, HSP90 might act as a downstream factor that fine-tunes long-term protein fate. Whereas previous studies have explored the neuroprotective potential of HSP70 and HSP90 modulators in stroke, such as geldanamycin analogs, arimoclomol, and celastrol, T817MA offers a distinct mechanism of action. Unlike these compounds, which primarily focus on HSP70 and HSP90 modulation, T817MA also specifically attenuates ER stress, which is a key feature of ischemic injury. This dual mechanism of suppressing ER stress while modulating the HSP70–HSP90 pathway sets T817MA apart from other HSP modulating agents. Furthermore, T817MA demonstrates significant neuroprotection without the off-target effects that are often observed with geldanamycin analogs, which can lead to systemic toxicity and challenges in clinical translation. The combined action of reducing ER stress and modulating the HSP70–HSP90 pathway makes T817MA a potentially promising therapeutic agent for ischemic stroke with fewer risks of side effects, thereby offering an advantage over some of the currently explored compounds in this class.

There are a few limitations to the present study. First, the use of the immortalized HT22 cells, while offering reproducibility and scalability, fails to fully recapitulate the complex microenvironment and multicellular interactions, such as glial crosstalk, vascular components, and immune responses, which occur during ischemic stroke *in vivo*. Unlike primary cultured neurons, which exhibit mature synaptic networks, age-related metabolic profiles, and physiological stress responses, HT22 cells lack critical aspects of neuronal diversity, regional specificity, and aging phenotypes (Pfeiffer et al., 2014). Second, our use of the pharmacological inhibitor, the HSP70 inhibitor PES, rather than genetic knockout techniques, introduces a greater risk of off-target effects and transient or incomplete target modulation. Small-molecule inhibitors often lack absolute specificity, thus potentially confounding results through the modulation of parallel pathways. Third, more *in vivo* experiments using siRNA transfection or knockout animals are needed to further confirm the molecular mechanisms underlying T817MA-induced protection observed in this study. Although T817MA demonstrates promising neuroprotective effects in preclinical models, several challenges remain for its clinical translation. First, the dosage and timing of administration that were observed to be effective in our rodent models may require significant adjustment for human therapy. Additionally, long-term safety and efficacy studies are needed to assess potential off-target effects and chronic toxicity. Addressing these concerns requires further detailed investigations and optimization of dosing regimens alongside the development of safe and effective delivery systems for T817MA.

## 5 Conclusion

In conclusion, HSPs serve as pivotal regulators of stroke pathophysiology, offering dual-edged yet tractable targets for neuroprotection. Our present study identified T817MA as a neuroprotective agent against ischemic stroke in both *in vitro* and *in vivo* experimental models. These protective effects were associated with reduced oxidative stress and ER stress, which were mediated through the regulation of the HSP70–HSP90 signaling pathway.

## Data Availability

The original contributions presented in the study are included in the article/Supplementary Material; further inquiries can be directed to the corresponding author.
